# Protein Quality of African Locust Bean—A High-Value Gathered Tree Food Contributing Protein and Palatability to Plant-Based Diets

**DOI:** 10.1155/2024/1596212

**Published:** 2024-08-27

**Authors:** Eliot T. Masters, Bokary Allaye Kelly

**Affiliations:** ^1^ Applied Business Nelson Marlborough Institute of Technology (NMIT), Nelson, New Zealand; ^2^ Programme Ressources Forestières Centre Régional de la Recherche Agronomique de Sikasso Institut d'Economie Rurale (IER), Sikasso, Mali

**Keywords:** flavor compounds, nitrogen conversion factor, palatability, plant proteins, umami

## Abstract

The African locust bean tree *Parkia biglobosa* (Jacq.) R.Br. ex G. Don is a leguminous species native to the Sudanian parkland of western Africa. The seed obtained from pods collected from trees by rural women is fermented into a dense and aromatic paste known as *soumbala*, *dawadawa*, or *iru*—a protein-rich condiment underlying much of West African cuisine, its rich umami base lending a depth of flavor as a “meat substitute” in plant-based diets for which animal protein is a limiting component. Here, we assess the protein quality of *P. biglobosa* seed and its fermented product (*soumbala*) from three locations of southern Mali comprising three different eco-climatic zones, to determine whether variation in nutritional composition and protein quality could be correlated with the geographic variables of latitude and elevation. Proximate composition was determined, and amino acid profiles were compiled for 19 amino acids, with results compared by location and eco-climatic zone. A protein quality test was conducted in the aggregate and for each zone using the WHO/FAO Protein Digestibility-Corrected Amino Acid Score (PDCAAS) method. Principal component analysis (PCA) was used to assess patterns of amino acid compositional variation between the three origins. The results underline the nutritional significance of African locust bean as a source of dietary protein and of a depth of flavor providing enhanced palatability to plant-based diets. Although the PCA biplot for the amino acid profiles does indicate geographic clustering, the variation in nutritional composition and protein quality is insignificant for the raw seed, but highly significant for the fermented product (*soumbala*). The results indicate no correlation between phytochemical parameters and geographic variables of latitude and elevation, suggesting that management and processing may contribute more to nutritional quality than product provenance. Further studies should assess specific processing methods and the ambient microbiome as factors contributing to protein quality.

## 1. Introduction

The African locust bean tree *Parkia biglobosa* (Jacq.) R.Br. ex G. Don is a valued nutritional resource across the western African savannah. The leguminous species occurs across the Sudanian biogeographical region extending from Senegal to South Sudan, a wooded savanna (or “dry forest”) on relatively fertile luvisols and cambisols with annual rainfall of 800–1500 mm [1]. Often found in association with the more widely known shea butter tree *Vitellaria paradoxa* C.F.Gaertn, *P. biglobosa* is a characteristic species of the parkland agroforestry system [2, 3], a traditional management practice characterized by protection of useful tree species when clearing fallow land, resulting in a mosaic landscape of cultivation and woodland. Over time, the selective conservation of individual trees with attributes favored by farming households has amounted to a state of “semidomestication” in some species [2–4]. The nutritional, livelihood, and economic value of parkland resources and the landscape-level environmental services they provide has attracted considerable research interest in recent decades, though attempts to quantify the value of wild and semidomesticated plant genetic resources within such systems have been limited [5], particularly with regard to their nutritional value and its intraspecies variation [6, 7].

After flowering (the double-lobed, or biglobose, flower accounting for its species name), the tree bears long fruit pods—the locust bean yielding a dry edible pulp and a seed rich in proteins and essential lipids which is traded widely across regional markets. The seed is not consumed directly but is fermented by ambient *Bacillus* species under alkaline conditions into a strong-smelling paste known as *soumbala* in the Francophone countries and *dawadawa* or *iru* elsewhere, the base of soups, stews, and the regional rice dishes *thieboudienne*, *riz gras*, and jollof rice.

When the testa is removed from the hard dry seed, it is easily fermented, a task undertaken by rural women involving an initial boil of 24 h or more, dehulling of the seeds, and a second boiling of 1–2 h, followed by aerobic fermentation over 24–72 h under ambient temperatures of 25°C–35°C, following which the fermented product is air-dried and molded into balls [1, 8]. Through fermentation, the hydrolytic degradation of seed proteins into free amino acids [8] creates a “precursor-rich environment” from which flavors can be developed through subsequent reactions [9], lending a depth and complexity to protein-limited plant-based meals typical across the region.

In addition to a protein content on the order of 40%, *soumbala* also provides essential lipids including polyunsaturated fatty acids, as well as riboflavin, a B vitamin otherwise deficient in West African diets [10]. During fermentation, while seed lipids are largely preserved, proteolytic activity generates increasing quantities of free amino acids along with a pronounced ammoniac odor. Fermentation also involves bacterial digestion of carbohydrates by galactosidase and sucrase activity, and also synthesis of the B vitamin riboflavin, levels of which treble while thiamine and niacin (as well as ascorbic acid) are significantly reduced along with antinutritional factors which impede protein availability, for example, phytic acid and oxalic acid [11].

While the protein content and quality of the fermented product are of great nutritional importance to the rural communities in which it is collected [10, 12], its market value is primarily based upon its taste profile, as a powerful source of umami underlying traditional cuisine, lending savory depth to a diet based largely on cereal staples and other plant-based foods limiting or deficient in protein. Adeyeye [13] calls it “a low-cost meat substitute” used by low-income households as “the premium meat of the stew”—improving the palatability and nutritional density of meals in which both flavor and protein are limiting factors [14, 15].

Production estimates from the 1980s cited by Adeyeye [13] suggest a population of 10 million trees distributed along a band of vegetation extending from Senegambia to Chad and Cameroon, producing a quarter of a million tons of locust bean, yielding about 170 thousand tons of the fermented bean product—annual per capita regional consumption of which has been estimated to range from 700 g to over 3.6 kg, reflecting its significance as a source of dietary protein.

Locust bean is significant in regional trade, as has been evident since the earliest recorded regional observations. Travelling through Northern Nigeria in 1853, Heinrich Barth encountered a caravan of donkeys loaded with cakes of soumbala at Zurmi, near the present-day Niger border [1]. Respondents interviewed in 2012 by EM at regional markets across the central basin region of West Africa from Côte d'Ivoire and Burkina Faso to Ghana, Togo, Benin, and Nigeria described northern trade corridors serving the Sahel region and southern corridors serving the immense market demand of Nigeria, with over 5000 MT moving between 12 regional markets in 100 kg bags of raw seed, at prices ranging between $440 and $1600 per metric ton. These figures indicate a thriving secondary market in local production of the fermented product—known as soumbala, nététou, dawadawa (or daddawa), and iru within the countries and areas of destination.

Regional trade in the fermented product is likewise brisk, with demand often exceeding supply—perhaps most notably between the Malian production zone and consumers in Senegal. The demand for Malian *soumbala* is so great in Senegal that as local stocks are depleted, Malian traders may sell a *soumbala* derived from fermented soya instead—a product commonly known as *miso* in Japan [16]. The evocative aroma of *soumbala* is primary sensory characteristic of the local markets on which it is sold by artisanal producers, and the regional markets on which it is traded in bulk as the most important food condiment of the western Africa region ([17] in [9]).

In their comprehensive review of in locust bean nutritional composition data, Termote et al. [18] called for further studies on the variability of nutrient composition according to ecoregion (agroecological zone), in order to identify the drivers of variation in nutrient content by distinguishing the influence of environmental characteristics (including soil, climate, and seasonality) from the innate varietal traits of genotype or provenance. Of the papers included in that review was a single study of nutritional composition between multiple sample sites in northern Benin [19], in which the authors speculated about the influence of geography yet did not interpret their results according to any specific geographic variables or ecotypes.

In a study of geographic patterns of genetic diversity in *P. biglobosa* which identified three major geographic clusters, Lompo et al. [20, 21] found the highest intrapopulation diversity in the central West Africa subregion including south-western Mali, which they characterized as a “hotspot” of *P. biglobosa* genetic diversity and an area of focus for genetic resources conservation. Gaisberger et al. [22] found that *P. biglobosa* was under severe threat in adjacent areas of Burkina Faso, under increasing climatic and anthropic pressures.

Species diversity is widely assumed to reflect a latitudinal distribution. Rohde [23], Vázquez and Stevens [24], and Willig and Presley [25] provide a detailed typology of the relevant ecological variables along latitudinal gradients, including temperature, rainfall, and seasonality (as a function of the angle of the sun above the horizon), and ecological factors of competition and environmental harshness. Araújo and Costa-Pereira [26] applied a latitudinal gradient to intraspecies diversity in 156 populations of 76 vertebrate and invertebrate animal taxa ranging between 54°S and 69°N, finding an inverse relationship between diversity and latitude. In the study area, latitude is directly correlated to altitude along the south side of the Niger River basin.

A series of earlier papers [27–29] assessed phenotypic variation, vegetative growth, and productivity of *Parkia biglobosa* at three locations of southeastern Mali selected so as to be representative of three distinct ecotypes or agroecological zones: the North Sudanian (500–800 mm rainfall on heavy soils), where natural vegetation is under severe anthropic pressure; the South Sudanian (800–1100 mm) with deep alluvial soils under continuous cultivation and short fallow; and North Guinean zone with over 1100 mm of annual precipitation, the longest fallow and highest density of woody species, bordering the forest zone to the south. The altitude of the study sites increases from north to south, ranging from the North Sudanian zone (275 m) to the South Sudanian (310 m) and North Guinean (330 m).

In the earlier studies cited above, the results were assessed by agroclimatic zone with three classification levels according to ecotype and the land use factor with two levels (fields and fallows). Within the study area, fixed plots of 50 by 50 m were established within cultivated fields and on fallow lands at 18 locations, comprising six fixed plots per ecological zone. Within each plots, five mature *P. biglobosa* trees of or exceeding 10 cm in diameter were measured, marked, and geolocated and were subsequently monitored over three annual cycles from 2019 to 2021 with data collected on flowering and fruiting phenology and on the yield of fruits, grains, and pulp.

Kelly, Kouyaté, and Dembélé [29] found no significant correlation between morphological traits and agroclimatic zones, whereas Kelly, Kouyaté, and Dembélé [27] found significant correlation between climatic zone and growth parameters, though not along a climatic gradient. Kelly, Kouyaté, and Dembélé [28] found that the density of the species was highest in the North Guinean zone, with decreasing density from south to north.

Drawing upon this earlier work and ongoing studies in the production zone of Mali, here, we evaluate protein content, composition and quality of *P. biglobosa* seed, and its fermented product (*soumbala*), in order to determine whether variation in the nutritional composition and protein quality could be linked to geographic variables along a latitudinal axis.

## 2. Materials and Methods

Whole dried seed and whole fermented seed were sampled in each of the three agroclimatic zones, on basis of the presence and frequency of *P. biglobosa* on cultivated fields and on fallow lands and the level of interest in participation on the part of local farmers.

Product samples were obtained at the study sites established in 2019, comprising as follows: Somasso (Bla district) in the North Sudanian zone (latitude 12.858611°, longitude −5.6075°), Zanzoni (Koutiala district) in the South Sudanian zone (lat. 12.614444°, lon. −5.534722°), and Diou (Kadiolo district) in the North Guinean zone (lat. 10.596111°, lon. −5.975833°).

**Figure 1:**
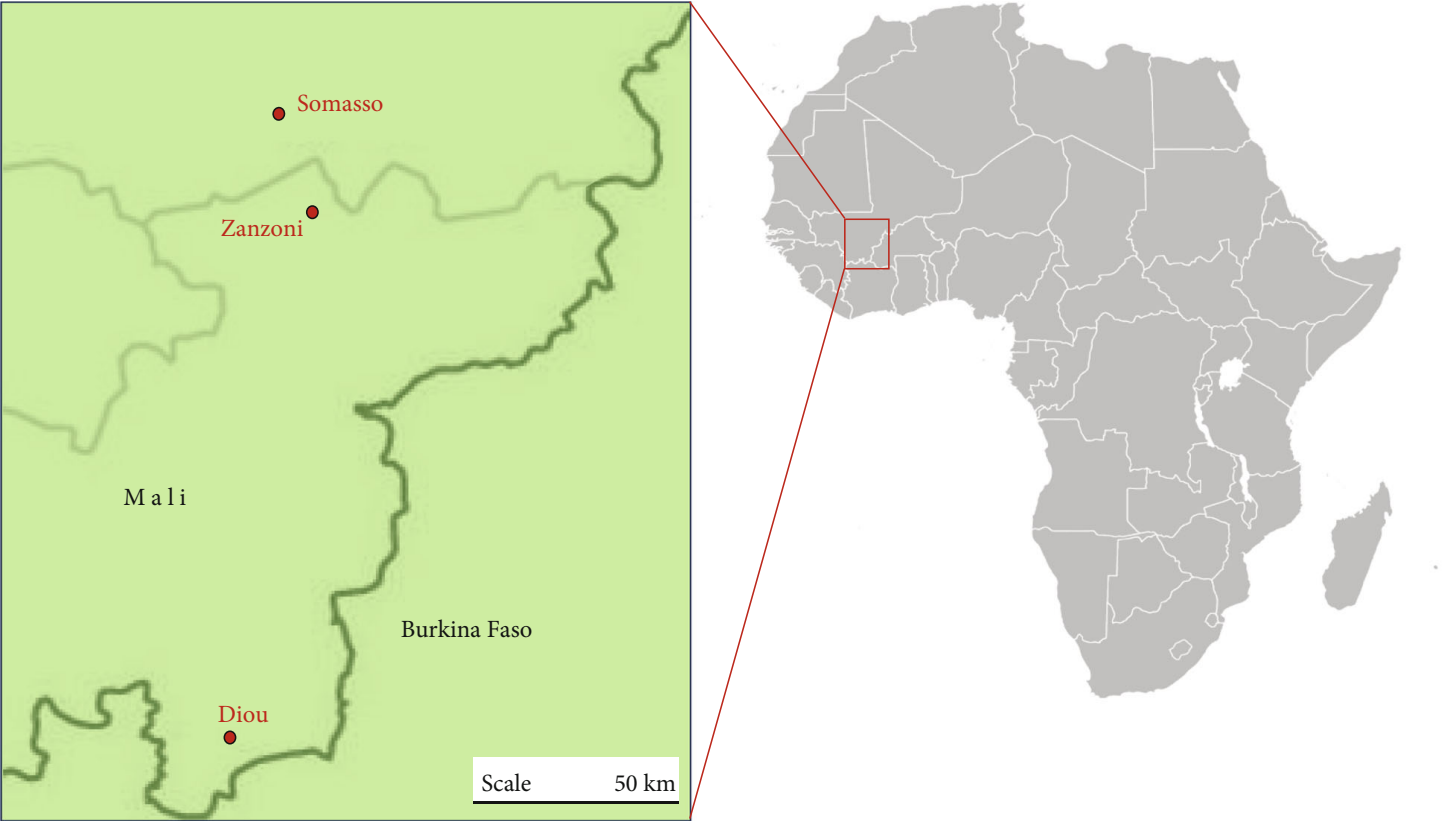
Sample locations.

Sample locations are indicated in Figure 1.

For the purpose of nutritional profiling, samples were obtained from three collaborating farmer households, consisting of three samples of dried seed and three samples of soumbala from each location. Seed samples reflect the combined harvest of a single household from multiple trees on one or more parcels (fields and fallows) under cultivation by, or otherwise accessible to, the respective farming household from which the sample originated. Samples of the fermented product likewise reflect the output of multiple trees on one or more parcels within the sampled location. Collaborating households were compensated for the samples according to current market value, at FCFA 1000 ($1.64) per kilogram of dried seed and FCFA 1500 ($2.48) per kilogram of soumbala.

All samples were dried in an electric oven at 50°C for 8 h and then milled to a fine powder using a Breville BCG 200 W grinder. Samples were packaged in clearly labelled cotton bags to avoid fungal growth and were sent by courier to analytical laboratories at the University of Nebraska in the United States for proximate nutritional analysis and to Macquarie University in Australia for amino acid profiling.

Proximate compositional profiles of all samples were obtained using AOAC official method 935.29 for moisture analysis, AOAC 985.01 for ash, and for protein AOAC 990.03 (combustion) using a nitrogen conversion factor of 6.25 for protein, in common with conventional practice for the species [18, 30]. Lipid extraction was undertaken using the FOSS Soxtec system according to manufacturer's suggestions, following which the carbohydrates were determined by calculation. Three replicate analyses were conducted for each test, and the results presented as an average (mean) with a standard deviation (SD) derived for each parameter.

Amino acid profiling analysis of all samples for all 20 proteinogenic amino acids including cysteine, tryptophan, hydroxyproline, and taurine was performed as per AOAC official methods 994.12 (amino acids in feeds), 982.30 (protein efficiency ratio), AOAC 988.15 (tryptophan), and AOAC 985.28 (sulfur amino acids), with minor modifications.

All samples were subjected to liquid hydrolysis by immersion in 6 M hydrochloric acid for 24 h at 110°C, whereby glutamine is converted into glutamic acid and asparagine is converted into aspartic acid; thus, the amounts of those acids reported here represent the total of their respective components. Following hydrolysis, labelling of amino acids was done using a Waters AccQ-Tag Ultra chemistry kit according to supplier recommendations and then analyzed using a Waters ACQUITY Premier Ultra-Performance Liquid Chromatography (UPLC) system. All samples were analyzed in duplicate, with results here expressed by average of the two replicate values.

A protein quality test was conducted using the WHO/FAO Protein Digestibility-Corrected Amino Acid Score (AAS) (PDCAAS) method, known by the acronym PDCAAS [31].

 $$\begin{array}{c} \text{PDCAAS}=\frac{\text{mg of limiting amino acid in }1\text{ g of test protein}}{\text{mg of limiting amino acid in }1\text{ g of reference protein}}\times \text{TD} \end{array}$$

where TD represents the true digestibility of the test protein, as determined by a rat fecal assay.

This test was applied using estimated TD values derived from Yakubu et al. [32, 33], which found in vitro protein digestibility of 65.91 g of hydrolyzed protein per 100 g protein in the raw seed and 74.56 g/100 g protein in the fermented product using the pepsin-pancreatin digestion method [33].

Analysis of variance (ANOVA) was applied to all analytical results in order to determine the relative significance of variance within and between the sample sites (zones) and whether any patterns of variation thus obtained could be associated with the geographic variables of latitude and elevation.

Tukey's HSD (honestly significant difference) test was used to assess the degree of variation between zones, and the null hypothesis (implying no difference between the population means for all pairs) was tested by the Tukey–Kramer test [34]. Significantly different values within a given parameter are indicated by the lowercase letters provided alongside value in parameters found to be significantly different [35], and SD is provided in all compositional tables and figures.

As a means of bringing a visual dimension to the complex patterns of variation within and between the amino acids profiles, diversity between sampled parameters was assessed using the principal component analysis (PCA) methodology, whereby complex data sets are reduced to the most diverse variables [36].

## 3. Results

### 3.1. Proximate Composition

Table 1 indicates the proximate composition of all samples by zone, showing insignificant difference between sites for the seed, but significant difference between sites for the fermented product (*soumbala*). In aggregate, the results indicate approximately 27 g per 100 g of protein in the seed and 39 g/100 g (with greater variation) in the fermented product, while the lipid content is approximately 14.5 g/100 g in the seed and over 40 g/100 g in the fermented product, presumably reflecting loss of carbohydrates through bacterial digestion during fermentation [1].

ANOVA results on composition show no statistical difference for any parameter for the raw seed, but significant difference (at a 95% confidence level) in the fermented product for protein (*p* = 0.00008724), lipids (*p* = 0.02244), and ash (*p* = 0.0009992).

The South Sudanian site of Zanzoni at the geographic median in latitude and elevation was found to have the highest protein and lowest lipid content, with no difference found between the other two sites, between which lie at the geographic extremes of the study area. For protein content, pairwise comparison of Zanzoni and Diou shows a *p* value of 0.0007893, between Zanzoni and Somasso (in closest geographic proximity) a *p* value of 0.0001552, and between Somasso and Diou (the most geographically distant sites) a *p* value of 0.7965, indicating no correlative relationship between protein content and the geographic variables of latitude and elevation.

### 3.2. Amino Acid Profiles

**Figure 2:**
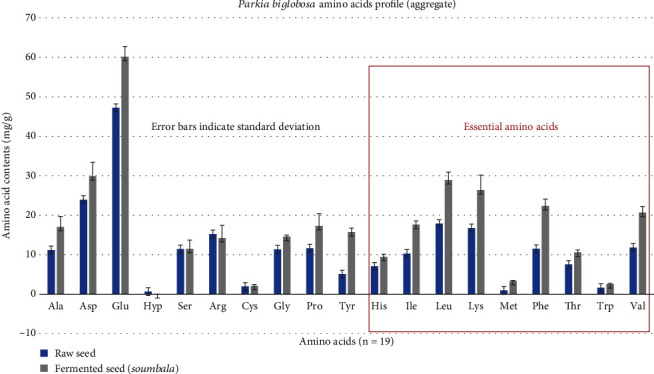
Amino acid profiles (aggregate).

Figure 2 presents the aggregate amino acid profiles of the raw *P. biglobosa* seed and the fermented product (soumbala). Aside from arginine, all other amino acids are significantly higher in the fermented product, including all of the amino acids classified as nutritionally essential.

Table 2 provides the amino acid profiles of the raw seed and fermented seed (soumbala) samples by site (zone), with significant difference in most amino acids found between locations in the soumbala profiles.

Although no value for methionine was reported for the seed sample B-2 from the North Sudanian site of Somasso, an amount of methionine present in the sample was detected, but due to factors such as variable oxidation or matrix effects, a robust quantitation could not be achieved. As a result, the mean methionine value for that location presented here was obtained as an average of the two samples for which methionine was reported.

The variation between the sites is not statistically significant at a 95% confidence interval for any amino acid in the raw seed aside from alanine, a minor component and a nonessential amino acid. By contrast, variation in amino acid content is generally significant in the fermented product, aside from the essential amino acid cysteine and the nonessential glutamic acid and glycine.

### 3.3. Protein Quality Assessment

**Figure 3:**
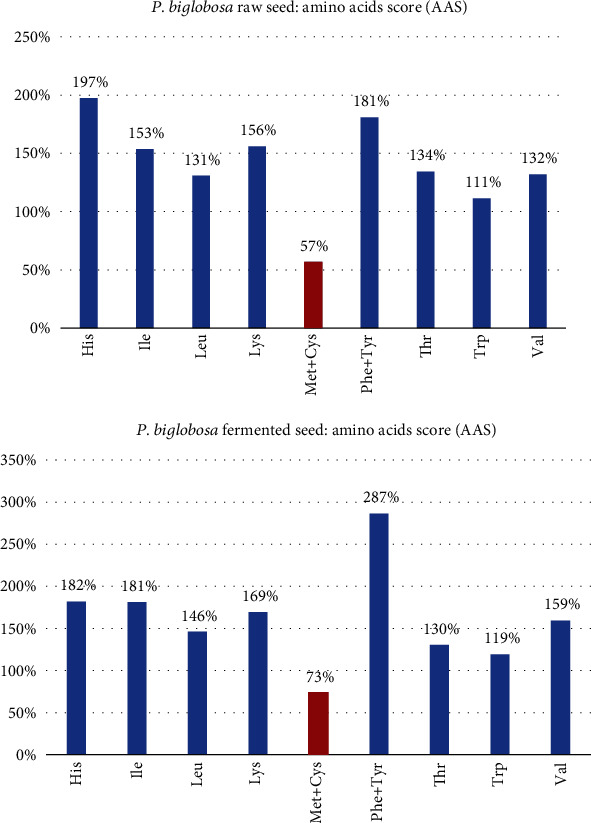
Aggregate amino acid scores (AAS).

As indicated in Figure 3, of all the essential amino acids quantified, both methionine and cysteine were limiting in both the raw seed and the fermented product (soumbala), resulting in AAS of 57% in the raw seed and 73% in the fermented product.

Table 3 indicates the protein quality for all samples of the raw seed and the fermented product (soumbala) as determined by the PDCAAS method, the limiting amino acids being methionine and cysteine (indicated in italics).

Table 4 indicates protein quality variation by zone as determined by the PDCAAS method, indicating no statistical difference between the seed samples, but highly significant difference in fermented seed protein quality between the three zones.  Table 5 illustrates the depth of this variation by paired comparison between sample sites, indicating that protein quality is significantly higher in the North Guinea zone and significantly lower in the South Sudanian zone.

### 3.4. PCA

**Figure 4:**
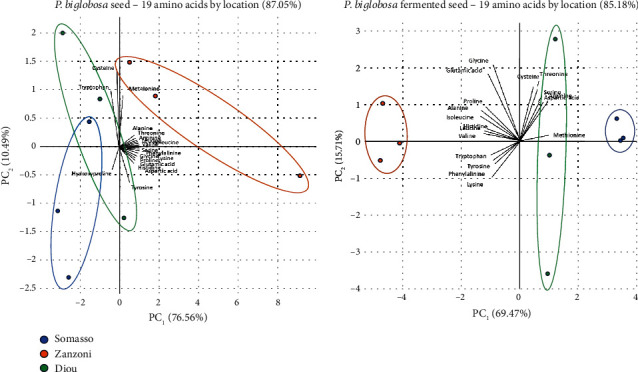
PCA results summary.

Figure 4 provides a visual representation of diversity and similarity between the amino acid profiles of all sample sites, underlining the outlying position of the centrally located South Sudanian site of Zanzoni, as compared to the closer association between the more distant sites of Somasso and Diou, which lie at the latitudinal extremes of the study area.

The PCA results underline the outlier status of the samples from the latitudinal median location of Zanzoni as compared to those from Somasso and Diou, which display greater similarity in amino acid profiles for the seed and soumbala (more notably for the latter), although they originate from opposite extremes of the sampling axis.

## 4. Discussion

The results indicate no correlation between the assessed compositional parameters and the variable of latitude and as such are comparable to the findings of earlier studies of the vegetative and productive phenology of the associated agroforestry species *Vitellaria paradoxa* across the Sudanian parkland of southern Mali [37, 38]. In those studies, samples were obtained along a diagonal gradient extending from across southern Mali between 11°7.777 N 8°22.263 W and 14°06.48 03 N 3°30.246 W. As in our results, those studies found no correlation to the geographic variables of latitude, noting greater variation among parameters between more proximate sites, with the central South Sudanian site of Mperesso an outlier in the total seed lipids, fatty acid profiles, and tocopherol content, much as Zanzoni has been identified here as an outlier in protein and lipid content. Taken together, these results imply the influence of biotic and abiotic factors as yet undetermined which cannot be reduced to the known parameters associated with latitude.

By comparison to the proximate values obtained for the raw seed, the content of moisture, ash, and carbohydrate is reduced in the fermented product by 25%, 35%, and 80%, respectively. Although fermented product samples did not necessarily originate in the same trees represented in the seed samples, the analysis is broadly indicative of the compositional changes brought about through preparation of the raw seed (including heating and removal of the seed coat) and during fermentation. It has been documented in earlier studies that during fermentation, the seed carbohydrate content is reduced by half as the component sugars are taken up by bacteria, thus increasing the proportion of protein and lipids accordingly, while these components are subsequently broken down into free amino acids and fatty acids [1].

The protein content values as determined by compositional analysis appear consistently greater than the total proteinogenic amino acid content of all samples, which amounts to about 83% of the proximate values in both the seed (at 22.4 against 27 g/100 g) and in the fermented product (at 32.4 against 39.2 g/100 g). It is not clear why the values appear to diverge so significantly, since the analysis covered only 19 amino acids, including both the proteinogenic amino acids and the nonproteinogenic nitrogen-containing compounds [39], and furthermore, it seems likely that some amino acids would have been lost during the hydrolysis stage. However, the simplest explanation would be that the conventional nitrogen conversion factor of 6.25 (implying 16 g of N per 100 g protein) is unrealistically high, noting that recent studies have indicated true NCF values within the range of 5.54–5.7 [40–42]. It should be noted that an NCF of 5.3 (as originally proposed by the University of Nebraska laboratory as a generic grain protein value unconnected with the species in the literature) would have resulted in a protein content of 22.88 g/100 g ± 0.51 with an SD of 1.35 for the seed and 33.26 g/100 g ± 0.393 with an SD of 1.04 for the fermented product.

The nonessential amino acid hydroxyproline (a minor compound in the raw seed) was found not to be present in the fermented product. Given that hydroxyproline is a major component of cell wall glycoproteins in some leguminous species [43, 44], this loss may be explained by the removal of the seed coat during prefermentation processing, noting further that the levels present in the seed (a mean value of 0.66 mg/g) are not far above the limit of reporting (LOR) of 0.2 mg/g.

In the aggregate, processing and fermentation results in a significant improvement in protein quality, from a PDCAAS value of 37% for the raw grain, to 54% in the fermented product, methionine and cysteine nutritionally limiting in both products. The PDCAAS of 37% for the raw seed is comparable to black bean, whereas the PDCAAS of 54% for soumbala is comparable to fava bean, kidney bean, or conventional soya bean [45].

While the nutritional value of soumbala is the focus of this study, it must be recognized that consumer and market demand is based on its sensory and organoleptic attributes—the aroma and taste which provide savor and palatability to vegetable dishes [46]. The distinct aroma profile of soumbala derives from the hydrolysis of the seed proteins and lipids during alkaline bacterial fermentation into ammonium (2%), free amino acids (10%), and short-chain fatty acids [8, 47] with as many as 125 volatile organic compounds contributing to its distinctively pungent aroma [48]. The flavor compounds of soumbala include specific amino acids contributing to taste, which have been classified by Tseng et al. [49] as sweet or bitter (the former including serine, threonine, alanine, and glycine and the latter including methionine, phenylalanine, tryptophan, tyrosine, isoleucine, leucine, arginine, histidine, and valine).

However, the flavor profile of soumbala is dominated by just two amino acids, most notably glutamic acid (or its ionic form L-glutamate), constituting over 20% of amino acids present in all samples, and aspartic acid (over 10% in all samples, with a slight reduction in the fermented product), for which variation between samples by site was found to be insignificant.

Although glutamic acid is just 30% higher in the fermented product, this brings it from under half of the raw seed proteins (still the predominant amino acid by a factor of 2 over the second highest component) to over 60% of amino acid content in the fermented product. Glutamic and aspartic acids have long been recognized as the “building blocks” of the umami taste characteristic of meats, poultry, and fish [50, 51] and also found in hard cheeses, mushrooms, and seaweed [49]. Chandrashekar et al. [52] classify umami as the savory taste sensation (alongside sweet, bitter, sour, and salty), and it is precisely this savory property for which the fermented product is valued by consumers. Used as as a "meat substitute" contributing a depth of flavor to the protein-inadequate plant-based diets of the poor [53], fermented locust bean increases the palatability of foods otherwise lacking in the umami taste characteristic of the more expensive (and flavorful) animal proteins.

Moreover, these compounds are not mere elements of flavor but have been found to play a role in protein nutrition. In the words of Yamaguchi and Ninomiya [54], palatability is a crucial nutritional factor which “promotes the selection, intake, absorption and digestion of foods” by involving all of the senses, but primarily taste. Kawai, Uneyama, and Miyano [55] refer to glutamic acid as a “signal” compound in the protein nutrition pathway, noting that it occurs human milk in concentrations exceeding the threshold requirement for umami taste. Uneyama et al. [56] observed that glutamate is active in the gastrointestinal tract and also in the liver, as a signal compound involved in modulating protein ingestion, digestion, and metabolism.

Yamaguchi and Ninomiya [54] profile free glutamic acid in 47 foods, of which “Soumbara/locust beans (West Africa)” shows the highest content, at 1700 mg/100 g—higher even than Parmigiano Reggiano (parmesan cheese) at 1680 mg/100 g. The values obtained here are significantly higher still, with a mean glutamic acid content of over 4400 mg/100 g in the seed and over 6000 mg/100 g in the fermented product. Our results align with those of earlier studies including Oke and Umoh [57] as cited in [1] for the seed (19.5 g/100 g protein, or 4388 mg/100 g) and with Oluwanyi and Bazambo 2016 for the fermented product (17.36 g/100 g protein, or 5625 mg/100 g). While it is difficult to speculate on why the values presented by Yamaguchi and Ninomiya [54] are so much lower, it is possible that their method did not involve hydrolyzation of glutamine into glutamic acid as per our methods.

Ham [59] contends that the perceived palatability of the fermented product has declined in recent decades, reflecting a shift in consumer taste away from the characteristic odors of the traditional product, driven by increasing consumer demand for the bouillon cubes industrially manufactured by multinational companies as an umami base for soups and stews, using patented processes specifically drawn from the traditional artisanal methods of African locust bean fermentation to produce a comparable taste effect with a reduced aroma and higher marketability [9]. With a view to preserving the culinary heritage of fermented African locust bean, recent studies have explored biotic and procedural interventions aimed at reducing its characteristic odor as a means of maintaining market share of the traditional product alongside the deracinated industrial offering [60].

### 4.1. Limitations

Data on soil composition, site-specific temperature, and rainfall could not be obtained for all three sample locations, and so it was not possible to assess the potential influence of these possibly relevant variables on the compositional parameters assessed here.

Furthermore, sampling limitations excluded an assessment of intraspecies diversity, and comparison between the composition of raw seed and the fermented seed samples cannot be made directly, since the fermented samples were not derived specifically from the raw seeds sampled in each location and thus did not necessarily originate in the same trees represented in the seed samples. Future studies could sample individual trees within each location and arrange local processing of seed samples into the fermented product under ambient conditions to better elucidate the effect of genetic, technical, and environmental factors on compositional changes obtained by fermentation.

From the nutritional perspective, the results presented here are based on secondary estimates of protein digestibility and as such do not address the actual availability of protein, nor of specific amino acids. Although soumbala has been valued as a source of lysine, which is deficient in the regional staple grains sorghum and millet [10], other accounts suggest that heat treatment during the processing of legume seeds effectively renders the lysine unavailable [61]. Further research on in vivo digestibility and availability would thus be merited.

Although the study identifies the umami flavor precursors glutamic and aspartic acids as most abundant all samples, no other compounds relevant to flavor were studied here, and as such, it should be noted that a complexity of factors contributes to the flavor profile, which derives from the protein hydrolysis of proteins into free amino acids as well as peptides and volatile compounds including aldehydes, ketones, and pyrazines [62]. Frøst et al. [63] describe the contribution of volatile compounds such as methylbutanal to the umami taste of certain seaweeds and refer to an “odor-taste congruency” enhancing the umami taste (or flavor), whereby the role of odor might be used systematically to create more palatable foods. Given that processing methods and the microecology of the fermenting product vary significantly and are known to result in a diversity of secondary compounds contributing to the aggregate flavor profile [64], future studies should likewise assess variation in these compounds, which might be further correlated to consumer organoleptic preferences.

## 5. Conclusions

The results indicate that the phytochemical parameters assessed here cannot be linked to geographic variables of latitude and elevation along the latitudinal axis. Given the skewed distribution of the three sample sites (27 km latitudinal distance between Somasso and Zanzoni and 224 km latitudinal distance between Zanzoni and Diou), it would be expected that compositional variation between the three would show greater similarity between Somasso and Zanzoni, with Diou more of an outlier based on geographic variables of latitude and altitude—but results clearly indicate otherwise.

It was found that differences in the raw seed composition, amino acids, and in protein quality by zone were not statistically significant at 95% confidence. However, differences in composition, amino acids, and protein quality of the fermented product by zone were highly significant. The significance of difference between the fermented product samples by zone may possibly reflect compositional differences in the seed which were insignificant in our data at 95% confidence, or variation in the fermentation process, including processing methods extant in and between the sample locations as well as local and regional diversity of the ambient microbiome during fermentation.

The compositional results imply an inverse relationship between the protein and the lipid content, while amino acid profiles and PDCAAS results indicate a similar relationship between the total protein content and protein quality. Samples from the South Sudanian site of Zanzoni were found to contain the highest protein content (and total amino acids), while samples from the North Guinean site of Diou were found highest in protein quality of the fermented product.

In common with earlier studies on associated parkland species, the impact of a latitudinal climatic gradient on phytochemical content was not proven by this study, which implies that processing methods and genetic factors (both macro- and micro-) are more important as drivers of variation.

The results of this study provide insights on the effects of artisanal fermentation on the macronutrient content of the fermented seed and quantify the protein quality and nutritional value of the traditional product—underlining the importance of the species as a livelihood and economic resource for the rural communities, who manage the trees and the landscapes on which they occur.

Taken together, it is hoped that these results might serve to valorize the species and promote its development and conservation as a regional nutritional resource contributing to systemic resilience in an uncertain future.

## Figures and Tables

**Table 1:** Proximate composition, *P. biglobosa* raw seed, and fermented seed (*soumbala*).

	**Somasso (NS)**		**Zanzoni (SS)**		**Diou (NG)**		**Between sites**			**p** ** value**
	**Mean**	**Std. dev.**	**Mean**	**Std. dev.**	**Mean**	**Std. dev.**	**Sum of squares SS**	**Mean square MS**	**F** **-stat**	
Seed (g per 100 g)										
Moisture	10.71	0.34	10.47	0.51	10.62	0.46	0.27	0.14	0.70	0.509
Ash	4.58	0.67	4.13	0.62	4.30	0.70	0.95	0.48	1.08	0.356
Protein	26.81	1.89	27.17	1.49	26.96	1.55	0.57	0.29	0.10	0.901
Lipids	14.67	0.74	14.68	1.06	14.26	1.22	1.02	0.51	0.49	0.620
Carbohydrates	43.22	2.59	43.56	1.85	43.85	1.62	1.76	0.88	0.21	0.815
Soumbala (g per 100 g)										
Moisture	7.27	0.39	9.07	0.73	7.69	0.58	15.98	7.99	23.74	0.000
Ash	2.42	0.42	2.86	0.24	3.08	0.30	2.01	1.00	9.34	0.001
Protein	38.47^b^	0.16	40.47^a^	0.59	38.74^b^	1.37	21.20	10.60	14.15	0.000
Lipids	41.74^a^	1.77	38.82^b^	3.11	41.94^a^	2.39	55.18	27.59	4.47	0.022
Carbohydrates	10.09	1.74	8.79	4.02	8.55	2.57	12.47	6.24	0.72	0.495

*Note:* Means without letters and means sharing the same letter are not significantly different at a significance level (*α*) of 0.5.

**Table 2:** Amino acid profiles by site (zone) with standard deviation (SD) and significance of difference (*p* value).

**Amino acid (mg/g)**	**Seed**							**Soumbala**						
	**Somasso (NS)**		**Zanzoni (SS)**		**Diou (NG)**		**p** ** value**	**Somasso (NS)**		**Zanzoni (SS)**		**Diou (NG)**		**p** ** value**
	**Mean**	**SD**	**Mean**	**SD**	**Mean**	**SD**		**Mean**	**SD**	**Mean**	**SD**	**Mean**	**SD**	
Alanine	10.72^b^	0.09	11.86^a^	0.66	10.96^b^	0.23	0.03	15.48^b^	0.12	20.36^a^	0.15	15.31^b^	2.02	0.003
Aspartic acid	23.14	0.81	25.41	1.75	23.27	1.32	0.15	32.86^a^	0.36	25.71^b^	0.86	31.043^a^	2.86	0.006
Glutamic acid	46.25	2.35	50.88	4.18	44.54	1.68	0.09	59.58	0.62	62.46	0.57	58.45	3.72	0.149
Hydroxyproline	0.75	0.08	0.57	0.08	0.65	0.07	0.07	—	—	—	—	—	—	—
Serine	11.05	0.11	12.08	0.79	11.13	0.23	0.07	13.87^a^	0.09	9.10^b^	0.24	11.42^ab^	1.77	0.004
Arginine	14.60	0.43	16.35	1.19	14.76	0.50	0.06	17.67^a^	0.22	10.82^b^	0.26	14.17^ab^	2.40	0.003
Cysteine	1.87	0.24	2.06	0.25	1.95	0.18	0.60	2.17	0.07	1.72	0.04	2.39	0.48	0.065
Glycine	11.07	0.14	11.83	0.54	11.17	0.22	0.07	14.41	0.08	14.76	0.42	14.61	0.56	0.597
Proline	11.36	0.17	12.37	0.88	11.26	0.29	0.09	16.51^ab^	0.17	20.85^a^	0.19	14.58^b^	2.44	0.004
Tyrosine	5.17	0.35	5.19	0.29	4.94	0.46	0.68	14.86^b^	0.10	17.03^a^	0.10	15.32^b^	0.32	0.000
Histidine	6.88	0.13	7.48	0.52	6.85	0.21	0.10	8.90^b^	0.07	10.32^a^	0.17	9.12^b^	0.24	0.000
Isoleucine	9.92	0.12	10.93	0.83	10.09	0.24	0.10	16.77^b^	0.04	18.69^a^	0.25	17.42^b^	0.76	0.006
Leucine	17.23	0.21	18.97	1.36	17.37	0.35	0.07	26.53^c^	0.02	30.98^a^	0.37	29.20^b^	0.96	0.000
Lysine	16.17	0.31	17.80	1.37	16.32	0.37	0.10	21.86^c^	0.18	30.45^a^	0.22	26.82^b^	1.51	0.000
Methionine	0.56	0.04	0.71	0.11	0.68	0.04	0.16	3.25^b^	0.01	3.23^b^	0.05	3.53^a^	0.08	0.001
Phenylalanine	11.09	0.14	12.22	0.96	11.19	0.20	0.10	20.41^c^	0.13	24.35^a^	0.27	22.3^b^	0.49	0.000
Threonine	7.17	0.11	7.96	0.55	7.45	0.15	0.07	11.14^a^	0.08	9.90^b^	0.22	10.70^ab^	0.64	0.022
Tryptophan	1.66	0.10	1.63	0.07	1.64	0.10	0.93	2.37^b^	0.05	2.76^a^	0.14	2.52^b^	0.01	0.004
Valine	11.41	0.10	12.48	0.88	11.56	0.24	0.10	18.79^c^	0.05	22.39^a^	0.23	20.81^b^	0.51	0.000
Total AAs	217.90	4.85	238.78	15.83	217.78	5.74	0.07	317.45	2.06	335.87	3.56	319.73	16.66	0.120

*Note:* Means without letters and means sharing the same letter are not significantly different at a significance level (*α*) of 0.5.

**Table 3:** Protein quality by PDCAAS (aggregate values).

**EAA**	**g of EAA in 100 g of tested food**	**mg of EAA in 1 g of tested protein**	**mg of EAA in 1 g of ideal protein**	**AAS**	**PD**	**PDCAAS**
Raw seed protein quality evaluation—PDCAAS						
His	0.71	31.57	16	197	65	
Ile	1.03	46.05	30	153	65	
Leu	1.79	79.73	61	131	65	
Lys	1.68	74.85	48	156	65	
*Met + Cys*	*0.29*	*13.09*	*23*	*57*	*65*	*0.37*
Phe + Tyr	1.66	74.13	41	181	65	
Thr	0.75	33.61	25	134	65	
Trp	0.16	7.35	6.6	111	65	
Val	1.18	52.77	40	132	65	
Total	9.253 g	413 mg	291 mg	1253	—	
Soumbala protein quality evaluation—PDCAAS						
His	0.94	29.11	16	182	75	
Ile	1.76	54.34	30	181	75	
Leu	2.89	89.11	61	146	75	
Lys	2.64	81.33	48	169	75	
*Met + Cys*	*0.54*	*16.74*	*23*	*73*	*75*	*0.54*
Phe + Tyr	3.81	117.47	41	287	75	
Thr	1.06	32.61	25	130	75	
Trp	0.25	7.86	6.6	119	75	
Val	2.07	63.71	40	159	75	
Total	15.967 g	492 mg	291 mg	1447	—	

*Note:* Aggregate values for methionine and cysteine are italicized here in order to reflect their status as limiting amino acids in both products.

**Table 4:** PDCAAS by location.

**Zone**	***P. biglobosa* raw seed**						***P. biglobosa* fermented seed (soumbala)**					
	**Sample 1**	**Sample 2**	**Sample 3**	**Mean**	**Std. dev.**	**Std. error**	**Sample 1**	**Sample 2**	**Sample 3**	**Mean**	**Std. dev.**	**Std. error**
Somasso (NS)	0.345	0.234	0.293	0.29	0.0553	0.0319	0.563	0.555	0.536	0.56^b^	0.0043	0.0025
Zanzoni (SS)	0.338	0.286	0.364	0.33	0.0397	0.0229	0.473	0.480	0.488	0.48^c^	0.0075	0.0043
Diou (NG)	0.377	0.306	0.345	0.34	0.0356	0.0205	0.585	0.630	0.600	0.61^a^	0.0229	0.0132
All (*n* = 9)				0.32						0.55		

*Note:* Means without letters and means sharing the same letter are not significantly different at a significance level (*α*) of 0.5.

**Table 5:** Tukey HSD/Tukey–Kramer paired comparison on *soumbala* protein quality (PDCAAS) variation.

**Pair**	**Difference**	**SE**	**Q**	**Lower CI**	**Upper CI**	**Critical mean**	**p** ** value**	**Group**	**SS**	**NG**
NS-SS	0.080	0.008165	9.798	0.04457	0.1154	0.03543	0.001092	NS	0.08	0.045
NS-NG	0.045	0.008165	5.5113	0.00957	0.08043	0.03543	0.01878	SS	0	0.13
SS-NG	0.125	0.008165	15.3093	0.08957	0.1604	0.03543	0.00009075			

## Data Availability

The authors affirm the availability of the original data upon which the study is based, which will be shared by request with interested researchers.

## References

[1] Hall J. B., Tomlinson H. F., Oni P. I., Buchy M., Aebischer D. P. (1997). Parkia biglobosa: a monograph. *School of agricultural and forest sciences publication*.

[2] Pullan R. A. (1974). Farmed parkland in West Africa. *Savanna*.

[3] Boffa J. M. (1999). *Agroforestry Parklands in Sub-Saharan Africa*.

[4] Seignobos C. (1982). Matières grasses, parcs et civilisations agraires (Tchad et Nord-Cameroun). *Les cahiers d'outre-mer*.

[5] Dawson I. K., Leakey R., Clement C. R. (2014). The management of tree genetic resources and the livelihoods of rural communities in the tropics: non-timber forest products, smallholder agroforestry practices and tree commodity crops. *Forest Ecology and Management*.

[6] Burlingame B., Charrondiere R., Mouille B. (2009). Food composition is fundamental to the cross-cutting initiative on biodiversity for food and nutrition. *Journal of Food Composition and Analysis*.

[7] Jamnadass R., Place F., Torquebiau E. (2013). Agroforestry, food and nutritional security. *ICRAF working paper no. 170*.

[8] Ouoba L. I. I., Rechinger K. B., Barkholt B., Diawara B., Traoré A. S., Jakobsen M. (2003). Degradation of proteins during the fermentation of African locust bean (*Parkia biglobosa*) by strains of *Bacillus subtilis* and *Bacillus pumilus* for production of soumbala. *Journal of Applied Microbiology*.

[9] Beaumont M. (2002). Flavouring composition prepared by fermentation with *Bacillus* spp. *International Journal of Food Microbiology*.

[10] Campbell-Platt G. (1980). African locust bean (*Parkia* species) and its west African fermented food product, dawadawa. *Ecology of Food and Nutrition*.

[11] Eka O. U. (1980). Effect of fermentation on the nutrient status of locust beans. *Food Chemistry*.

[12] Reddy N. R., Pierson M. D., Sathe S. K., Salunkhe D. K., Beuchat L. R. (1983). Legume-based fermented foods: their preparation and nutritional quality. *Critical Reviews in Food Science & Nutrition*.

[13] Adeyeye E. I. (2013). The effect of fermentation on the dietary quality of lipids from African locust bean (*Parkia biglobosa*) seeds. *Elixir Food Science*.

[14] Rackis J. J., Sessa D. J., Honig D. H. (1979). Flavor problems of vegetable food proteins. *Journal of the American Oil Chemists' Society*.

[15] Roland W. S., Pouvreau L., Curran J., van de Velde F., de Kok P. M. (2017). Flavor aspects of pulse ingredients. *Cereal Chemistry*.

[16] Bass H. H. (2012). *Mali’s Agro-Industry: A SWOT-Analysis*.

[17] Odunfa S. A., Oyewole O. B. (1986). Identification of *Bacillus* species from ‘iru’, a fermented African locust bean product. *Journal of Basic Microbiology*.

[18] Termote C., Odongo N. O., Dreyer B. S., Guissou B., Parkouda C., Vinceti B. (2022). Nutrient composition of *Parkia biglobosa* pulp, raw and fermented seeds: a systematic review. *Critical Reviews in Food Science and Nutrition*.

[19] Urua I. S., Uyoh E. A., Ntui V. O., Okpako E. C. (2013). Effect of processing on proximate composition, anti-nutrient status and amino acid content in three accessions of African locust bean (*Parkia biglobosa* jacq.) benth. *International Journal of Food Sciences and Nutrition*.

[20] Lompo D., Vinceti B., Konrad H., Gaisberger H., Geburek T. (2018). Phylogeography of African locust bean (Parkia biglobosa) reveals genetic divergence and spatially structured populations in West and Central Africa. *Journal of Heredity*.

[21] Lompo D., Vinceti B., Gaisberger H. (2017). Genetic conservation in Parkia biglobosa (Fabaceae: Mimosoideae)-what do we know?. *Silvae Genetica*.

[22] Gaisberger H., Kindt R., Loo J., Schmidt M., Bognounou F., Da S. S. (2017). Spatially explicit multi-threat assessment of food tree species in Burkina Faso: a fine-scale approach. *PLoS One*.

[23] Rohde K. (1992). Latitudinal gradients in species diversity: the search for the primary cause. *Oikos*.

[24] Vázquez D. P., Stevens R. D. (2004). The latitudinal gradient in niche breadth: concepts and evidence. *The American Naturalist*.

[25] Willig M. R., Presley S. J., Della Sala D. A., Goldstein M. I. (2018). Latitudinal gradients of biodiversity: theory and empirical patterns. *Encyclopedia of the Anthropocene*.

[26] Araújo M. S., Costa-Pereira R. (2013). Latitudinal gradients in intraspecific ecological diversity. *Biology Letters*.

[27] Kelly B. A., Kouyaté A. M., Dembélé S. G. (2022). Leaf and fruit characteristics of *Parkia biglobosa* (Jacq.) Benth according to agro climatic zones and land use in southern Mali. *Research in Plant Biology*.

[28] Kelly B. A., Kouyaté A. M., Dembélé S. G. (2022). Effect of climatic zone and land use practices on pod, seed and pulp yield of *Parkia biglobosa* in southern Mali. *International Journal of Scientific Research Updates, 2022*.

[29] Kelly B. A., Kouyaté A. M., Dembélé S. G. (2021). Variation of *Parkia biglobosa* morphological traits according to land use and agro-climatic zones in southern Mali. *African Journal of Plant Science*.

[30] Adeyeye E. I. (2006). Amino acids composition of fermented African locust bean (*Parkia biglobosa*). *Journal of Applied and Environmental Sciences December*.

[31] Schaafsma G. (2005). The protein digestibility-corrected amino acid score (PDCAAS)—a concept for describing protein quality in foods and food ingredients: a critical review. *Journal of AOAC International*.

[32] Yakubu C. M., Sharma R., Sharma S. (2022). Fermentation of locust bean (*Parkia biglobosa*): modulation in the anti-nutrient composition, bioactive profile, in vitro nutrient digestibility, functional and morphological characteristics. *International Journal of Food Science & Technology*.

[33] Yakubu C. M., Sharma R., Sharma S., Singh B. (2022). Influence of alkaline fermentation time on in vitro nutrient digestibility, bio- & techno-functionality, secondary protein structure and macromolecular morphology of locust bean (*Parkia biglobosa*) flour. *LWT–Food Science and Technology*.

[34] Jaccard J., Becker M. A., Wood G. (1984). Pairwise multiple comparison procedures: a review. *Psychological Bulletin*.

[35] Piepho H. P. (2018). Letters in mean comparisons: what they do and don’t mean. *Agronomy Journal*.

[36] Morris E. K., Caruso T., Buscot F. (2014). Choosing and using diversity indices: insights for ecological applications from the German Biodiversity Exploratories. *Ecology and Evolution*.

[37] Kelly B. A., Davrieux F., Bouvet J.-M. (2018). A shea butter rich in tocopherols (vitamin E) at the Dogon plateau and Seno Bankass in Mali (West Africa). *Journal of Phytology*.

[38] Kelly B. A., Davrieux F., Piombo G., Kamissoko S., Senou O. (2014). Variation des constituants chimiques du beurre de *Vitellaria paradoxa* (karité) en fonction du gradient climatique Nord-Sud au Mali. *Les Cahiers de l'Economie rurale*.

[39] Shen G., Fan X., Yang Z., Han L. (2016). A feasibility study of non-targeted adulterant screening based on NIRM spectral library of soybean meal to guarantee quality: the example of non-protein nitrogen. *Food Chemistry*.

[40] Angell A. R., Mata L., de Nys R., Paul N. A. (2016). The protein content of seaweeds: a universal nitrogen-to-protein conversion factor of five. *Journal of Applied Phycology*.

[41] Mossé J. (1990). Nitrogen-to-protein conversion factor for ten cereals and six legumes or oilseeds. A reappraisal of its definition and determination. Variation according to species and to seed protein content. *Journal of Agricultural and Food Chemistry*.

[42] Sosulski F. W., Imafidon G. I. (1990). Amino acid composition and nitrogen-to-protein conversion factors for animal and plant foods. *Journal of Agricultural and Food Chemistry*.

[43] Cassab G. I., Nieto-Sotelo J., Cooper J. B., van Holst G. J., Varner J. E. (1985). A developmentally regulated hydroxyproline-rich glycoprotein from the cell walls of soybean seed coats. *Plant Physiology*.

[44] Vanetten C. H., Miller R. W., Earle F. R., Wolffe I. A., Jones Q. (1961). Plant protein constituents, hydroxyproline content of seed meals and distribution of the amino acid in kernel, seed coat, and pericarp. *Journal of Agricultural and Food Chemistry*.

[45] Boye J., Wijesinha-Bettoni R., Burlingame B. (2012). Protein quality evaluation twenty years after the introduction of the protein digestibility corrected amino acid score method. *British Journal of Nutrition*.

[46] Shahidah A. A., Farouq A. A., Magashi M. A., Ibrahim A. D. (2019). Taste profile and consumer preference of “dawadawa” produced from the seeds of Parkia biglobosa, Glycine max and Hibiscus sabdariffa. *International Journal of Biological and Chemical Sciences*.

[47] Esse M. Y., Guehi T. S., Grabulos J. (2021). Fate of proteic and lipidic compounds during production of a traditional legume condiment (soumbala) made from African locust bean (*Parkia biglobosa*) seeds. *International Journal of Food Science & Technology*.

[48] Ouoba L. I. I., Diawara B., Annan N. T., Poll L., Jakobsen M. (2005). Volatile compounds of soumbala, a fermented African locust bean (*Parkia biglobosa*) food condiment. *Journal of Applied Microbiology*.

[49] Tseng Y. H., Lee Y. L., Li R. C., Mau J. L. (2005). Non-volatile flavour components of *Ganoderma tsugae*. *Food Chemistry*.

[50] Yamaguchi S. (1979). The umami taste. *Boudreau*.

[51] Mouritsen O., Styrbæk K. (2014). *Umami: Unlocking the Secrets of the Fifth Taste*.

[52] Chandrashekar J., Hoon M. A., Ryba N. J., Zuker C. S. (2006). The receptors and cells for mammalian taste. *Nature*.

[53] Sop M. M. K., Fotso M., Gouado I., Tetanye E., Zollo P. A. (2008). Nutritional survey, staple foods composition and the uses of savoury condiments in Douala, Cameroon. *African Journal of Biotechnology*.

[54] Yamaguchi S., Ninomiya K. (2000). Umami and food palatability. *The Journal of Nutrition*.

[55] Kawai M., Uneyama H., Miyano H. (2009). Taste-active components in foods, with concentration on umami compounds. *Journal of Health Science*.

[56] Uneyama H., Gabriel A. S., Kawai M., Tomoe M., Torii K. (2008). Physiological role of dietary free glutamate in the food digestion. *Asia Pacific Journal of Clinical Nutrition*.

[57] Oke O. L., Umoh I. B. (1978). Lesser known oilseeds. 1. Chemical composition. *Nutrition Reports International, 1978*.

[58] Oluwaniyi O., Bazambo I. O. (2016). Nutritional and amino acid analysis of raw, partially fermented and completely fermented locust bean (*Parkia biglobosa*) seeds. *African Journal of Food, Agriculture, Nutrition and Development*.

[59] Ham J. R. (2017). Cooking to be modern but eating to be healthy: the role of Dawa-Dawa in contemporary Ghanaian foodways. *Food, Culture & Society*.

[60] Amoa-Awua W. K., Awusi B., Owusu M. (2014). Reducing the atypical odour of dawadawa: effect of modification of fermentation conditions and post-fermentation treatment on the development of the atypical odour of dawadawa. *Food Control*.

[61] Ibiyemi S. A., Ehusani R. O., Amaorgu F. B., Atteh J. A. (1989). Studies of the thermal effect on *Parkia* seeds. *Food Chemistry*.

[62] Azokpota P., Hounhouigan J. D., Annan N. T., Nago M. C., Jakobsen M. (2008). Diversity of volatile compounds of afitin, iru and sonru, three fermented food condiments from Benin. *World Journal of Microbiology and Biotechnology*.

[63] Frøst M. B., Hartmann A., Petersen M. A., Duelund L., Mouritsen O. G. (2021). Odour-induced umami–olfactory contribution to umami taste in seaweed extracts (dashi) by sensory interactions. *International Journal of Gastronomy and Food Science*.

[64] Odunfa S. A. (1985). Biochemical changes in fermenting African locust bean (*Parkia biglobosa*) during ‘iru’ fermentation. *International Journal of Food Science & Technology*.

